# In Situ Monitoring the Potassium-Ion Storage Enhancement in Iron Selenide with Ether-Based Electrolyte

**DOI:** 10.1007/s40820-021-00708-1

**Published:** 2021-08-18

**Authors:** Xiaodan Li, Jinliang Li, Wenchen Zhuo, Zhibin Li, Liang Ma, Zhong Ji, Likun Pan, Wenjie Mai

**Affiliations:** 1 Siyuan Laboratory, Guangzhou Key Laboratory of Vacuum Coating Technologies and New Energy Materials, Guangdong Provincial Engineering Technology Research Center of Vacuum Coating Technologies and New Materials, Department of Physics, Jinan University, Guangzhou, Guangdong 510632 People’s Republic of China; 2 Shanghai Key Laboratory of Magnetic Resonance, School of Physics and Electronic Science, East China Normal University, Shanghai, 200241 People’s Republic of China

**Keywords:** Iron selenide, Ether-based electrolyte, In situ visualization technique, Potassium-ion batteries

## Abstract

As one of the promising anode materials, iron selenide has received much attention for potassium-ion batteries (KIBs). Nevertheless, volume expansion and sluggish kinetics of iron selenide result in the poor reversibility and stability during potassiation–depotassiation process. In this work, we develop iron selenide composite matching ether-based electrolyte for KIBs, which presents a reversible specific capacity of 356 mAh g^−1^ at 200 mA g^−1^ after 75 cycles. According to the measurement of mechanical properties, it is found that iron selenide composite also exhibits robust and elastic solid electrolyte interphase layer in ether-based electrolyte, contributing to the improvement in reversibility and stability for KIBs. To further investigate the electrochemical enhancement mechanism of ether-based electrolyte in KIBs, we also utilize in situ visualization technique to monitor the potassiation–depotassiation process. For comparison, iron selenide composite matching carbonate-based electrolyte presents vast morphology change during potassiation–depotassiation process. When changing to ether-based electrolyte, a few minor morphology changes can be observed. This phenomenon indicates an occurrence of homogeneous electrochemical reaction in ether-based electrolyte, which results in a stable performance for potassium-ion (K-ion) storage. We believe that our work will provide a new perspective to visually monitor the potassium-ion storage process and guide the improvement in electrode material performance.

**Supplementary Information:**

The online version contains supplementary material available at 10.1007/s40820-021-00708-1.

## Highlights

- The iron selenide composite with ether-based electrolyte presents excellent potassium storage performance.
- We develop in situ visualization technique to monitor the potassiation–depotassiation process.
- Iron selenide composite in ether-based electrolyte presents a homogeneous electrochemical reaction, resulting in a stable potassium-ion storage.

**Supplementary Information:**

The online version contains supplementary material available at 10.1007/s40820-021-00708-1.

## Graphical abstract

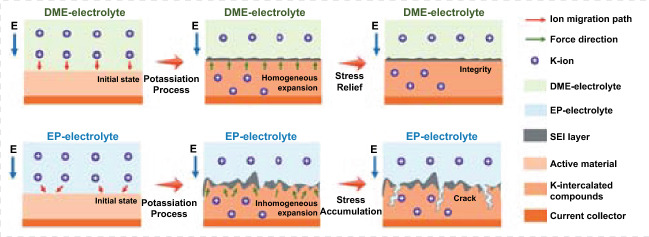

## Introduction

Since commercialization, lithium-ion batteries (LIBs) have developed rapidly and gained a monopoly position in portable device and electric vehicle industry due to the handiness and high energy density [1–4]. However, scarce reserves and maldistribution of lithium resource restrict the further development of LIBs in large-scale energy system [5–7]. Exploring low-cost energy storage system becomes urgent. As one of the promising alternatives, potassium-ion batteries (KIBs) have been regarded as an ideal energy storage system to replace LIBs due to the similar properties to lithium and abundant potassium resources [8–10]. Despite these advantages, the electrode materials usually accommodate large volume variations during the repetitive potassiation–depotassiation process due to the large size of potassium (K)-ion, which results in the irreversible behavior and degraded cycling stability [11, 12]. Therefore, seeking suitable host materials with high reversibility and stability for K-ion storage becomes an important research direction.

Because of the low cost and stability, enormous efforts are mainly focused on the carbonaceous materials, which have become the primary selectivity for KIBs [13–15]. However, low theoretical capacities of carbonaceous materials restrict the further improvement in the K-ion storage performance, forcing scientists to explore other high-performance host materials [16, 17]. Due to the superior K-ion storage performance, iron selenide also obtains lots of attention [18, 19], whereas enormous volume variations also persecute the improvement in the cycling stability for KIBs [20, 21]. Designing smart structures of iron selenide becomes an important path to address the issue of volume variations, which is helpful for the improvement in stability of KIBs. Liu et al. designed iron selenide anchored on nitrogen-doped carbon via an in situ chemical transformation method, achieving a reversible specific capacity of 434 mAh g^−1^ after 70 cycles at 100 mA g^−1^ for KIBs [22]. Zhao et al. synthesized iron selenide nanodots porous carbon networks by a gas-pumped self-expanding method, presenting a reversible specific capacity of 330 mAh g^−1^ after 100 cycles at 100 mA g^−1^ for K-ion storage [23]. Wang et al. obtained dispersed iron selenide nanoparticles in porous carbon nanofibers via a multistep hydrothermal method, retaining a decent charge capacity of 201 mAh g^−1^ after 50 cycles at 100 mA g^−1^ for KIBs [24]. Although the above work delivered the excellent K-ion storage performance, the complex preparation process is still unsatisfactory for the current demand of KIBs. Exploring simple methods to improve the electrochemical performance of iron selenide is urgent.

As another effective strategy, optimizing electrolyte can also remarkably enhance the K-ion storage performance of electrode [25–27]. Currently, ether-based electrolytes have been demonstrated to facilitate the formation of robust solid electrolytes interface (SEI) layer, which can provide a favorable working environment for K-ion transportation and contribute to the cycling stability. Wu et al. found that dimethyl ether electrolyte could hold the interface stability of SnSb/C anodes against large volume changes, eventually realizing an excellent cyclability [28]. Lei et al. obtained Bi anode cooperated with ether-based electrolyte for K-ion storage, achieving a fast kinetics and high tolerance of volume change, which contributed to the high K-ion storage performance [29]. Han et al. designed FeS_2_@C composite cooperated with ether-based electrolyte and raised the cutoff voltage, implementing a boosting long-term cycle stability [30]. In spite of these works, more material systems cooperated with ether‐based electrolyte still need to be further explored. Meanwhile, due to different reaction environments of electrodes in ether‐based electrolytes, some unconventional electrochemical reaction phenomena compared with that in carbonate-based (some named as ester-based) electrolytes occur and the detailed mechanism is still unclear. Utilizing in situ techniques to monitor the electrochemical reaction of electrodes and probing the underlying mechanism in ether‐based electrolytes have become necessary [31–33].

In this work, we rationally obtained reduced graphene oxide coating iron selenide (FeSe_2_@RGO) composite by a simple hydrothermal process. After optimization, we found that our FeSe_2_@RGO composite matching ether‐based electrolyte presented a remarkable improvement in electrochemical performance compared with that using the carbonate-based electrolyte. To explore the improvement in electrochemical performance, we also measured the mechanical properties of electrodes in both electrolytes. The result shows that the electrode in the ether-based electrolyte exhibits higher Young's modulus, which can effectively enhance the stability of electrode. Furthermore, the visualization technique was also developed, which could in situ monitor the electrochemical reaction process of electrode. It was found that the electrode in ether-based electrolyte presented homogeneous electrochemical reaction, resulting in a stable morphology change during potassiation–depotassiation process, which contributed to the improvement in electrochemical performance. Compared with the vast morphology change of electrode in carbonate-based electrolyte, a few minor changes could be observed, indicating an occurrence of homogeneous electrochemical reaction in ether-based electrolyte, which resulted in a stable performance for K-ion storage.

## Experiment

### Synthesis

Typically, GO was obtained by modified Hummers’ method from natural graphite, which has been reported in our previous work [34, 35]. For the FeSe_2_-RGO composites, 1.067 g Fe(NH_4_)_2_(SO_4_)_2_·6H_2_O and 2 g citric acid were dissolved into 30 mL deionized water, and then, 30 mL aqueous solution with 1 g NaBH_4_ and 0.431 g Se powder was dropped under vigorous stirring. To form homogeneous dispersions, moderate GO was added into above solution under stirring and sonication over 1 h. The mixture was transformed into a 100 mL Teflon-lined stainless steel autoclave and heated at 180 °C for 12 h. Subsequently, black precipitate was collected, washed with water for several times and dried under freezer dryer. After that, the black precipitate was annealed at 400 °C for 3 h under the flowing nitrogen to obtain the FeSe_2_@RGO composite. Among them, the composites added 0, 180, 240 and 300 mg GO were named as FeSe_2_, FeSe_2_@RGO-1, FeSe_2_@RGO and FeSe_2_@RGO-3, respectively. Unless otherwise specified, the electrode should be the FeSe_2_@RGO. For comparison, pure RGO was also obtained by the similar methods without the precursor of FeSe_2_.

### Characterization

The morphologies of samples were measured by field-emission scanning electron microscopy (FESEM, Zeiss) and transmission electron microscopy (TEM, FEI). The structures were confirmed by X-ray diffraction (XRD, D8 Rigaku9000) and Raman spectroscopy (Horiba T6400). The surface features were recorded by X-ray photoelectron spectroscopy (XPS, Thermo Fisher Scientific, K-Alpha). For the in situ visualization observation, we used metallographic microscope (Leica) cooperated with an electrochemical reaction cell to realize the in situ observation of electrode.

### Electrochemical Measurement

The as-prepared samples were used as the anode materials of KIBs to measure the electrochemical performance. The active material, super P and carboxylmethyl cellulose were mixed in the weight ratio of 8:1:1 in deionized water to form a uniform slurry. After that, the slurry was covered on Cu foil and dried overnight at 80 °C in a vacuum oven. Generally, the mass loading of above electrode was about 1.0–1.5 mg cm^−2^ and was acted as work electrode in CR2032 coin cells, which were assembled in argon-filled Etelux glovebox (Lab 2000) with the water and oxygen content less than 0.1 ppm. The K metal foil and Whatman glass fiber membrane were served as counter electrode and separator, respectively. The solution of ethylene glycol dimethyl ether with 1 M and 5 M potassium bis(fluorosulfonyl)imide (KFSI) was used as electrolyte, which was named as DME-1 and DME. For comparison, we also used 1 M KFSI in ethylene carbonate and propylene carbonate (1: 1, v/v) as electrolyte, which was named as EP. The galvanostatic charge–discharge (GCD) curves and the cycling performance were measured by battery test system (Neware BTS 4000) with the voltage ranging from 0.01 to 3 V at a current density of 100 mA g^−1^ unless otherwise specified. The cyclic voltammetry (CV) curves were recorded by electrochemical workstation (Shanghai ChenHua). The electrochemical impedance spectroscopies (EIS) were measured by Princeton electrochemical workstation (Veras STAT 3400). For the in situ visualization measurement, we used the dual electrode electrolytic tank under argon atmosphere, as shown in Fig. S1. Figure S2 shows the photograph of in situ visualization testing system. The detail assembly process is similar to the K-ion half battery. Firstly, the active material electrode was put in bottom and then placed the separator and metallic K foil which have been punched. The top was sealed with the quartz glass, which serves as observation window for the visualization. During the measurement, the in situ electrolytic tank was connected with the electrochemical station at a scan rate of 0.6 mV s^−1^. From the observation window, we could obtain the changes of electrode morphology during different potassiation–depotassiation states by optical microscope.

## Results and Discussion

### Materials Characterization

**Fig. 1:**
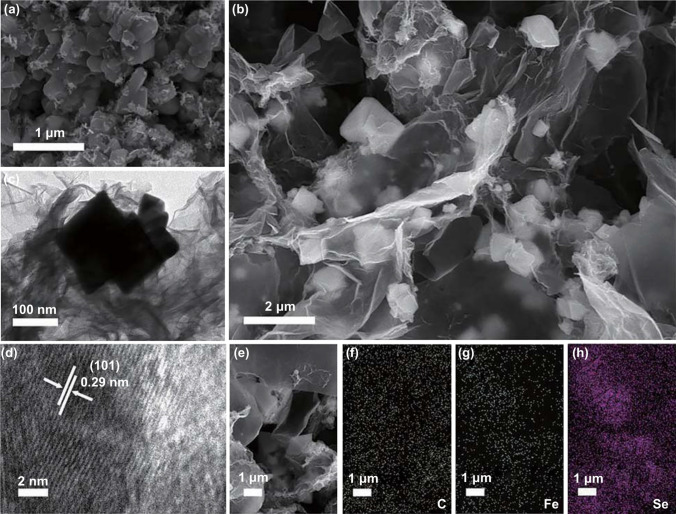
SEM images of **a** FeSe_2_ and **b** FeSe_2_@RGO, **c** low-resolution and **d** high-resolution TEM image of FeSe_2_@RGO. **e**–**h** C, Fe, Se elements mapping of FeSe_2_@RGO

Figure 1a shows the SEM image of FeSe_2_, which exhibits a typical granular structure. With the introduction of RGO in composite, the FeSe_2_ particles are packaged by RGO nanosheets to form a network structure, as shown in Fig. 1b. From the TEM image of FeSe_2_@RGO (Fig. 1c), the package structure of composite is also confirmed. Due to the excellent conductivity and stability of RGO in composite, the package network structure of composite can effectively enhance the electronic transport property and accommodate the volume expansion during potassiation–depotassiation process. Figure 1d shows the high-resolution TEM image of FeSe_2_@RGO. The distinct lattice fringes can be detected, indicating good crystallinity of FeSe_2_. A lattice distance of 0.29 nm is observed, which can be indexed to the (101) plane of FeSe_2_ (PDF No. 79-1892) [36, 37]. Figure 1e–h presents the element mapping of FeSe_2_@RGO composite. The result shows that Se and Fe are distributed uniformly in the FeSe_2_ particle and the C is distributed homogeneously in RGO layers. Figure S3a presents the XRD pattern of RGO, FeSe_2_ and FeSe_2_@RGO. We also found that FeSe_2_@RGO composite shows the same peaks compared with FeSe_2_ (PDF No. 79-1892), indicating that the introduction of RGO in composite does not change its structure [21]. No RGO peaks appear in composite, which should be due to the weak crystallinity of RGO [38]. To further investigate the structure of FeSe_2_@RGO composite, the Raman spectrum of FeSe_2_@RGO composite is provided (Fig. S3b). Two Raman peaks at low wavenumbers can be observed, which should be due to the rocking and stretching vibration of Se-Se bonds or their combination [39]. Other two peaks at high wavenumbers should be the D and G bands of RGO, further indicating that RGO has been introduced in the composite. To examine the surface property, XPS spectra of our composite were conducted. Figure S3c shows the Fe 2*p* high-resolution XPS spectrum. Three peaks can be deconvoluted at 707.3, 711.1 and 720.3 eV, which are ascribed to the Fe 2*p*_3/2_, satellite peak of Fe 2*p*_3/2_ and Fe 2*p*_1/2_, respectively [40]. Figure S3d presents the Se 3*d* high-resolution XPS spectrum, which can be deconvoluted into four peaks. Among them, two peaks at 55.2 and 55.8 eV correspond to Se 3*d*_5/2_ and Se 3*d*_3/2_, respectively [40].

### Electrochemical Performance Analysis

**Fig. 2:**
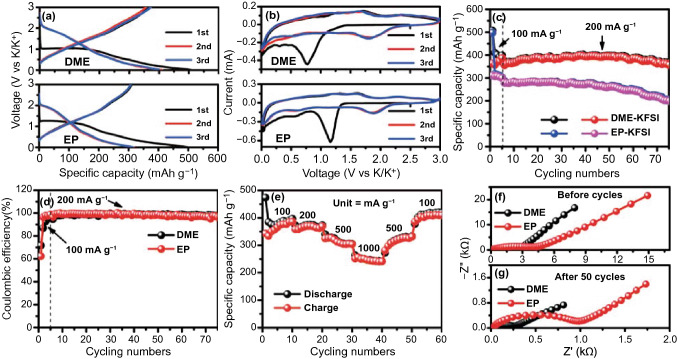
**a** GCD curves, **b** CV curves, **c** cycling numbers and **d** Coulombic efficiencies of FeSe_2_@RGO electrode with DME-based and EP-based electrolytes; **e** rate performance of FeSe_2_@RGO electrode with DME-based electrolyte; EIS spectra of FeSe_2_@RGO electrode with DME-based and EP-based electrolytes **f** before and **g** after 50 cycles

Figure S4 shows the cycling performance of RGO, FeSe_2_, FeSe_2_@RGO-1, FeSe_2_@RGO and FeSe_2_@RGO-3, and the results show that FeSe_2_@RGO exhibits better K-ion storage performance. Based on this, we selected FeSe_2_@RGO to match different electrolytes for analyzing the effect of electrolyte. To optimize the electrolyte, we also attempt to using DME-1 electrolyte in our electrode for KIBs. Figure S5 shows the corresponding GCD curves. It is found that the battery cannot charge to the set voltage of 3 V, indicating that the DME-1 is inappropriate for KIBs. Figure 2a shows the GCD curves of FeSe_2_@RGO with DME- and EP-based electrolytes. An irreversible reaction in initial potassiation process can be found in the electrode using both electrolytes. However, the electrode in DME-based electrolyte receives a high reversibility for KIBs, indicating the higher transfer ability of K-ion in DME-based electrolyte. To further investigate the electrochemical reaction, the CV curves of electrode matching DME-based and EP-based electrolytes are also provided, as shown in Fig. 2b. An obvious irreversible cathodic peak of battery with DME-based electrolyte at 0.77 V can be observed, which is attributed to the formation of SEI layer. But the irreversible cathodic peak of electrode in EP-based electrolyte exhibits a higher voltage (1.16 V), indicating that the EP-based electrolyte will decompose at a higher voltage to form SEI layer. In the subsequent cycles, all the CV curves exhibit similar shape, indicating that the batteries in both electrolytes exhibit the similar reaction process. Figure 2c shows the cycling performance of the batteries in DME-based and EP-based electrolytes. To facilitate the adequate electrochemical reaction of electrode material, we firstly measure the cycling performance at 100 mA g^−1^ after 5 cycles and then switched to the high current density of 200 mA g^−1^. We find that the FeSe_2_@RGO matching EP-based electrolyte only delivers an initial discharge specific capacity of 497 mAh g^−1^ at 100 mA g^−1^ and keeps an initial reversible specific capacity of 310 mAh g^−1^. After switching the current density to 200 mA g^−1^, our battery with EP-based electrolyte shows a distinct capacity decay to 198 mAh g^−1^ after 75 cycles. When changing the electrolyte to DME-based electrolyte, the electrode delivers a similar initial discharge specific capacity of 507 mAh g^−1^ at 100 mA g^−1^. However, the initial reversible specific capacity of 364 mAh g^−1^ shows a significant improvement. Meanwhile, it is also found that the electrode in DME-based electrolyte receives a specific capacity of 356 mAh g^−1^ after 75 cycles when the current density converts to 200 mA g^−1^, which exhibits higher capacity stability compared with the electrode in EP-based electrolyte. Furthermore, an initial Coulombic efficiency (CE) of the electrode in DME-based electrolyte (71.8%) can be observed, which is higher than that of electrode in EP-based electrolyte (62.4%, Fig. 2d), indicating that DME-based electrolyte will promote the reduction in irreversible products [41, 42]. To further investigate the electrochemical performance, we also present the rate performance of electrode in DME-based electrolyte and EP-based electrolyte, as shown in Figs. 2e and S6. It is found that our electrode in DME-based electrolyte delivers the reversible specific capacities of 386, 369, 304 and 242 mAh g^−1^ in the current densities of 100, 200, 500 and 1000 mA g^−1^, respectively, which is also higher than that of the electrode in EP-based electrolyte. With the recovery of current densities to 500 and 100 mA g^−1^, the reversible specific capacities also recover to 331 and 409 mAh g^−1^, respectively. This result indicates that our electrode in DME-based electrolyte presents excellent reversibility. To analyze the influence of different electrolytes for K-ion storage, the EIS spectra of electrode with DME- and EP-based electrolytes before and after cycles are provided (Fig. 2f, g). A noticeably smaller impedance arises when the electrode matches the DME-based electrolyte after the cycles, indicating that DME-based electrolyte can facilitate the K-ion transmission in battery [43]. In addition, we also find that the impedances of both electrodes decrease after cycles, which should be due to the activated process of electrode, resulting in the enhancement of ion migration. Figure S7 shows the SEM images of electrode in DME- and EP-based electrolytes after cycles. It is found that the electrode presents flatness in DME-based electrolyte and a crack can be observed in the electrode using EP-based electrolyte, demonstrating that DME-based electrolyte contributes to the stability of electrode.

### Mechanical Properties Analysis

**Fig. 3:**
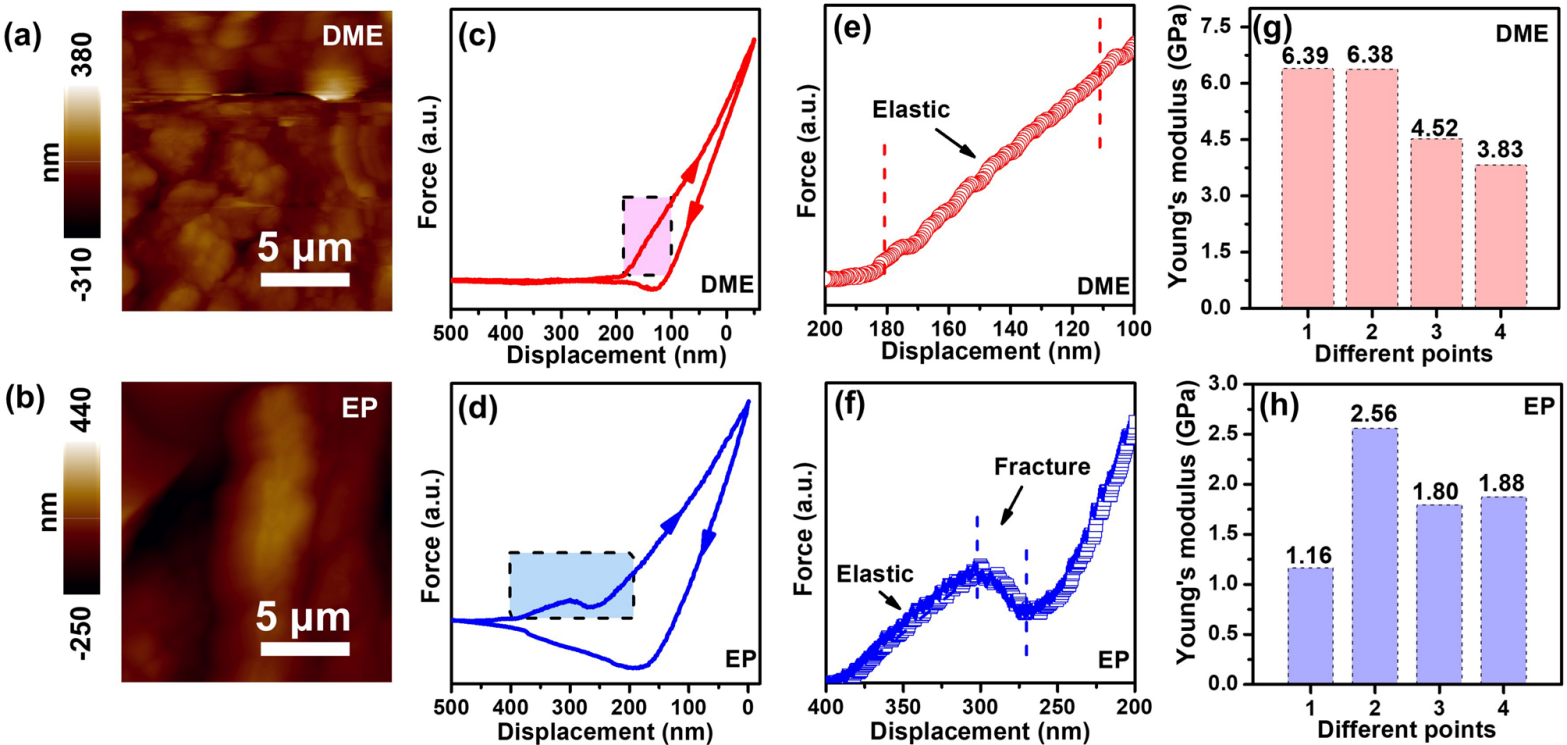
AFM images of FeSe_2_@RGO electrode using **a** DME-based and **b** EP-based electrolyte. Representative force–displacement curves FeSe_2_@RGO electrode using **c** DME-based and **d** EP-based electrolyte after 50 cycles. A detailed force analysis of SEI layers using **e** DME-based and **f** EP-based electrolytes after 50 cycles. Young's modulus of FeSe_2_@RGO electrode using **g** DME-based and **h** EP-based electrolytes

To further analyze the influence of different electrolytes for K-ion storage, the mechanical properties of both electrodes in DME- and EP-based electrolytes after cycles are provided. Figure 3a, b shows the AFM images of FeSe_2_@RGO electrodes in different electrolytes, in which the average roughness of the electrodes using DME- and EP-based electrolytes can be calculated to be 40 and 73 nm, respectively. This result indicates that DME-based electrolyte contributes to the stability of electrode during potassiation–depotassiation process. Figure 3c–f presents the force–displacement curves and corresponding detailed force analysis of SEI layers. In general, representative force–displacement curves are obtained from the press-in and pull-out process of the samples’ surface by the AFM tip. In the pull-in process, the AFM tip will instantaneously lose the force if the surface presents the fracture feature, which exhibits a sudden downward force. The force responses in DME-based electrolyte include linear elastic region by the response from electrode, indicating the existence of elastic surface of electrode [25, 44]. In contrast, the force responses in EP-based electrolyte include linear elastic region and slope change region. The slope change region was induced by perfectly plastic process or hardening process, possibly because of fracture and tip sliding [45]. The irreversible plastic deformation will ruin surface morphology, while elastic deformation contributes to reliving strain and maintaining integrity during potassiation–depotassiation process [25]. To quantify the mechanical properties, we calculate the Young’s modulus values of electrodes using these types of electrolytes (Fig. 3g, h). An average Young’s modulus value of 5.28 GPa of electrode using DME-based electrolyte can be received, which is 2.8 times larger than that of electrode using EP-based electrolyte (1.85 GPa). Based on the great change of Young’s modulus values, we suggest that the electrode will form compact and robust SEI layer in ether-based electrolyte. Such robust SEI layer is helpful for the inhibition of interfacial fracture, which facilitates the homogeneous electrochemical reaction for K-ion storage. On the contrary, lower Young’s modulus values of electrode in EP-based electrolyte indicate the formation of loose and unstable SEI layer, which will cause the unstable performance of electrode during potassiation–depotassiation process [44].

### In situ Visualization Analysis

**Fig. 4:**
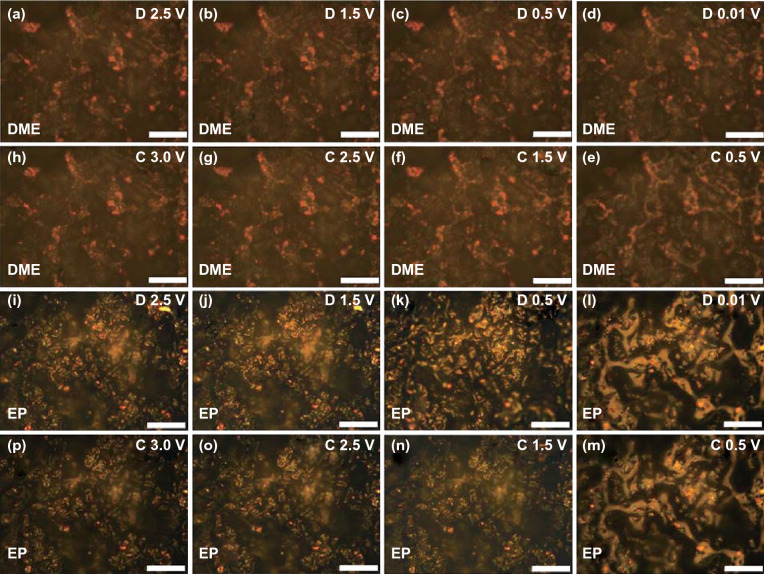
In situ visualization: **a**–**h** different potassiation–depotassiation states of FeSe_2_@RGO electrode using DME-based electrolyte in the first cycle; **i**–**p** different potassiation–depotassiation states of FeSe_2_@RGO electrode using EP-based electrolyte in the first cycle. The scale bar in each image is 50 μm

To further monitor the K-ion storage enhancement of electrode in different electrolytes, we developed the in situ visualization technique to observe the reaction process in real time. Figure 4a–h shows the visual images of electrode using DME-based electrolyte under different potassiation–depotassiation states in the first cycle. It is found that only a few particles on the electrode gradually strip together and the corresponding luster becomes slightly bright during the potassiation process. This phenomenon is due to the light reflection, which is caused by the volume expansion of the electrode during the insertion of K-ion. In the depotassiation process, it is found that the strip shape of electrode disappears and recovers to the original shape, indicating that our electrode presents an excellent reversibility. Generally, we consider that the expansion of the electrode material is closely related to the capacity in the same electrode. From the above results, the morphology of the electrode only presents a few minor changes, indicating that our electrode presents a homogeneous potassiation and depotassiation in DME-based electrolyte. According to the calculation in previous work, the K-ion presents relatively low energy change during migration process in the ether solvent, indicating that K-ion can migrate into electrode more easily in ether-based electrolyte [13, 46]. Therefore, we suggest that homogeneous potassiation and depotassiation process is mainly due to the high migration of K-ion in DME-based electrolyte. Furthermore, the homogeneous potassiation and depotassiation of electrode also provide less destruction of SEI layer, contributing to form a pyknotic SEI layer, which results in the high Young's modulus of SEI layer. In addition, the high Young's modulus of SEI layer also can resist the stress caused by local growth of electrode, which also facilitates the homogeneous potassiation and depotassiation reaction. To further confirm this result, we also provide the visual images of electrode using DME-based electrolyte under different potassiation–depotassiation states in the second cycle (Fig. S8a-h), which shows a similar result in the first cycle. Due to the homogeneous reaction of electrode in DME-based electrolyte, the integrity of electrode will be held during potassiation–depotassiation process, which contributes to the improvement in its cycling performance [47, 48]. When EP-based electrolyte is used (Fig. 4i–p), the phenomenon of morphologic change is even more drastic. Due to the relatively low migration of K-ion in carbonate solvent, a larger K-ion concentration difference occurs during potassiation–depotassiation process. This K-ion concentration difference will induce the inhomogeneous electrochemical reaction, resulting in the drastic morphologic change during potassiation–depotassiation process. In addition, the lower mechanical property of SEI layer in EP-based electrolyte may be beyond restraint excessive local stress, which also results in the enormous morphologic change. The visual images (Fig. S8i–p) in the second cycle also confirm the great variation in electrode during potassiation–depotassiation process. Due to the great variation in the subsequent cycles, the pulverization of electrode will be easier to occur, which results in the capacity decay of electrode. In addition, we also select the images of electrode in EP-based electrolyte with max variation during potassiation–depotassiation process for comparison, as shown in Fig. S9. Videos S1 and S2 show the whole reaction process of electrode using DME-based and EP-based electrolytes, respectively, in which the change of electrodes can be intuitively observed.

**Fig. 5:**
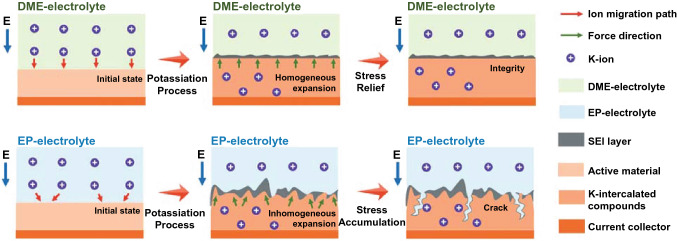
Schematic illustration of expansion process of electrodes in DME- and EP-based electrolyte

From the test of mechanical properties of SEI layer and the in situ visualization results of electrode, we raise the schematics to describe the expansion process of electrodes in DME-based and EP-based electrolytes, as shown in Fig. 5. Due to better ion mobility in ether-based electrolyte, it presents a shorter path for K-ion migration, which facilitates a homogeneous volume expansion of FeSe_2_@RGO electrode during the potassiation process. In addition, the robust SEI layer also provides a protection to control the expansion of the electrode material, which also results to the stress relief and realizes to integrity of electrode in subsequent potassiation–depotassiation process. If changing to EP-based electrolyte, it presents a longer path for K-ion migration due to the lower ion mobility in carbonate-based electrolyte. Therefore, an inhomogeneous volume expansion of electrode will occur, leading to a huge morphological change of electrode. Meanwhile, the low mechanical property of the SEI layer in EP electrolyte also results in the crack with the stress accumulation in subsequent potassiation–depotassiation process. This phenomenon will seriously damage the cyclic stability of electrode for K-ion storage.

### Dynamics Analysis

**Fig. 6:**
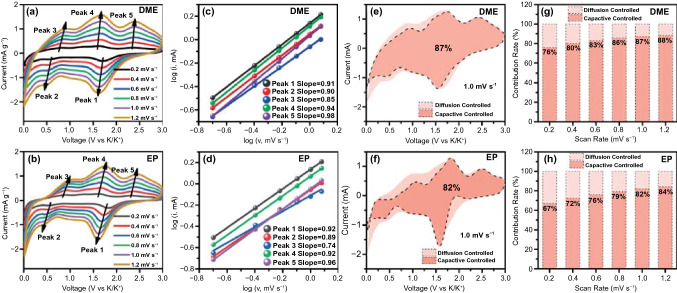
CV curves of FeSe_2_@RGO electrode using **a** DME-based and **b** EP-based electrolyte in different scan rates. The relationship between log (*i*) and log (*v*) of FeSe_2_@RGO electrode using **c** DME-based and **d** EP-based electrolytes. The capacitive contribution area of FeSe_2_@RGO electrode using **e** DME-based and **f** EP-based electrolytes at 1.0 mV s^−1^. Capacitive contribution ratios across different scan rates of FeSe_2_@RGO electrode using **g** DME-based and **h** EP-based electrolytes

Figure 6a, b shows the CV curves of FeSe_2_@RGO electrode using DME-based and EP-based electrolyte in different scan rates after 5 cycles. All the CV curves exhibit the similar shape, but the CV curves of electrode in DME-based electrolyte present broad redox peaks. To investigate the change of CV curves, we analyzed their electrochemical behavior. The relationship between peak current (*i*) and scan rate (*v*) is determined by Eq. (1) [49, 50]:

1 $$i=a{v}^{b}$$

which can be rearranged to Eq. (2):

2 $$\mathrm{log}i=\mathrm{log}a+b\mathrm{log}v$$

where *a* and *b* are the adjustable parameters and *b* is confirmed by the slope in Eq. (2). Usually, the *b* value closed to 0.5 is the diffusion-controlled behavior and the *b* value closed to 1 indicates the capacitive behavior. In Fig. 6c, d, it is found that the *b* values of all the peaks are closed to 1, illustrating that the electrode in both electrolytes exhibits capacitive behavior. To quantify the electrochemical behavior, we calculate the capacitive contributions of electrode in both electrolytes according to Eq. (3) [51, 52]:

3 $$i\left(V\right)={k}_{1}v+{k}_{2}{v}^{1/2}$$

which can be converted to Eq. (4):

4 $$i\left(V\right)/{v}^{1/2}={k}_{1}{v}^{1/2}+{k}_{2}$$

where *k*_1_*v* and *k*_2_*v*^1/2^ are deemed as the capacitive and diffusion-controlled behaviors, respectively. According to Eq. (4), the electrode in DME-based electrolyte receives a capacitive contribution of 87% at a scan rate of 1 mV s^−1^ (Fig. 6e), which is higher than that of electrode in EP-based electrolyte (82%, Fig. 6f). Figure 6g, h shows the capacitive contributions at different scan rates of electrode in DME-based and EP-based electrolytes, respectively. The electrode in DME-based electrolyte delivers the capacitive contributions of 76%, 80%, 83%, 86%, 87% and 88% at the scan rates of 0.2, 0.4, 0.6, 0.8, 1.0 and 1.2 mV s^−1^, respectively. Compared with the electrode in DME-based electrolyte, the electrode in EP-based electrolyte presents the lower capacitive contributions of 67%, 72%, 76%, 79%, 82% and 84% at the scan rates of 0.2, 0.4, 0.6, 0.8, 1.0 and 1.2 mV s^−1^, respectively. According to this result, we suggest that the capacitive contribution of electrode is closely related to the electrolyte. In DME-based electrolyte, K-ion will present high ion migration rate, indicating that K-ion can insert into electrode uniformly under the effect of electric field forces. The homogeneous insertion of K-ion contributes to the ion adsorption on the surface of electrode, which facilitates the improvement in capacitive contribution. Of course, the relatively higher capacitive contribution of electrode in DME-based electrolyte is also helpful for the improvement in cycling stability of electrode, which is consistent with the results of visualization measurement.

### In situ Raman Analysis

**Fig. 7:**
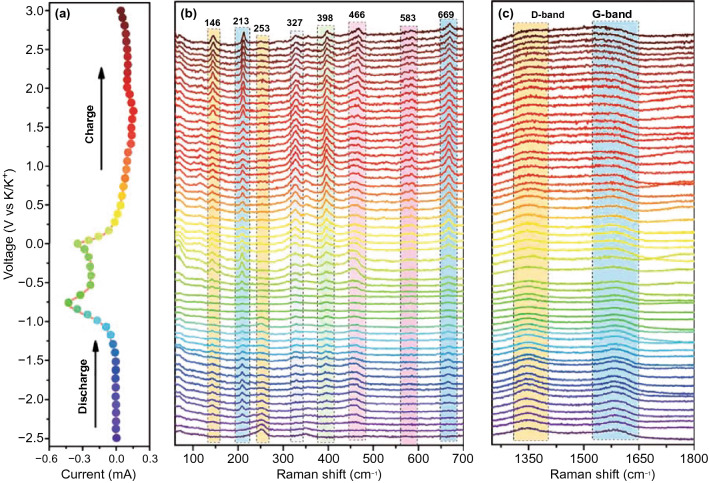
In situ Raman spectra of FeSe_2_@RGO electrode using DME-based electrolyte in different potassiation–depotassiation states: **a** the corresponding voltage position; in situ Raman spectra at **b** low wavenumbers and **c** high wavenumbers

Finally, we also provide in situ Raman spectra of electrode in DME-based electrolyte in Fig. 7 to further illustrate the potassiation–depotassiation process. Figure S10 displays the photograph of the practical in situ Raman spectra testing system. In the in situ Raman spectra, Fig. 7a shows the testing potassiation–depotassiation states according to the CV curves. Figure 7b, c presents the corresponding Raman spectra of electrode in low wavenumbers region and high wavenumbers region, respectively. In the initial state, a distinct peak at 253 cm^−1^ could be detected, which is attributed to the intrinsic peak of FeSe_2_. With the potassiation process proceeding, this peak gradually disappears at 1.8 V due to the break of the original vibration mode of FeSe_2_ and a new peak at 213 cm^−1^ appears, indicating that FeSe_2_ transforms into intermediate. We also notice that all peaks disappear in complete potassiation, which is related to the loss of resonance by the K-ion intercalant [53]. During the depotassiation process, some new peaks at 146, 327, 398, 466 and 669 cm^−1^ arise, and we suggest that this phenomenon should be the structural remodeling of FeSe_2_ intermediate rather than the primary structure of FeSe_2_. In the high wavenumber region, D-band and G-band can be observed in initial stage. With the potassiation process proceeding, we can detect a slight change of intensity of D-band and G-band. The intensity of G-band increases after completely depotassiation compared with that in the initial stage, indicating the formation of more orderliness of carbon in electrode, which is due to the realignment of interlayer distance [54].

## Conclusions

In this work, we rationally obtained iron selenide composite cooperated with different electrolytes for KIBs and the results show that the electrode matching ether-based (DME) electrolyte presents a reversible specific capacity of 356 mAh g^−1^ at 200 mA g^−1^ after 75 cycles, which is better than that cooperated with carbonate-based (EP) electrolyte. To investigate the electrochemical enhancement mechanism of KIBs, we also utilized in situ visualization technique and Raman spectra to monitor their potassiation–depotassiation process. The result shows that iron selenide presents less volume expansion in ether-based electrolyte. According to the test of mechanical properties of the electrode in both electrolytes, an elastic interphase layer in ether-based electrolyte can be detected, which also contributes to the improvement in reversibility and stability for KIBs and is consistent with the results from the in situ characteristics.

## Supplementary Information

Below is the link to the electronic supplementary material.

Supplementary file1 (PDF 679 kb)

Supplementary file2 (MP4 9929 kb)

Supplementary file3 (MP4 10023 kb)
