# Supplementary material for: In Situ Monitoring the Potassium-Ion Storage Enhancement in Iron Selenide with Ether-Based Electrolyte
Source: Nanomicro Lett. 2021 Aug 18;13:179. doi: 10.1007/s40820-021-00708-1 (PMC8374025; doi:10.1007/s40820-021-00708-1)
Supplement: Supplementary file 1 — Supplementary file1 (PDF 679 kb) [file 40820_2021_708_MOESM1_ESM.pdf]

Supporting Information for

In-Situ Monitoring the Potassium Ion Storage Enhancement in Iron Selenide with Ether-Based Electrolyte

Xiaodan Li[1], Jinliang Li[1,*], Wenchen Zhuo[1], Zhibin Li[1], Liang Ma[1], Zhong Ji[1], Likun Pan[2], Wenjie Mai[1,*]

[1]Siyuan Laboratory, Guangzhou Key Laboratory of Vacuum Coating Technologies and New Energy Materials, Guangdong Provincial Engineering Technology Research Center of Vacuum Coating Technologies and New Materials, Department of Physics, Jinan University, Guangzhou, Guangdong 510632, P. R. China

[2]Shanghai Key Laboratory of Magnetic Resonance, School of Physics and Electronic Science, East China Normal University, Shanghai 200241, P. R. China

*Corresponding authors. E-mail: lijinliang@email.jnu.edu.cn (Jinliang Li); wenjiemai@email.jnu.edu.cn (Wenjie Mai)

Supplementary Tables and Figures

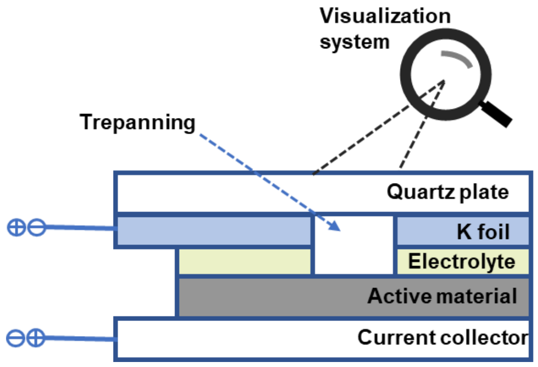

Fig. S1 Detail testing schematic of in-situ visualization

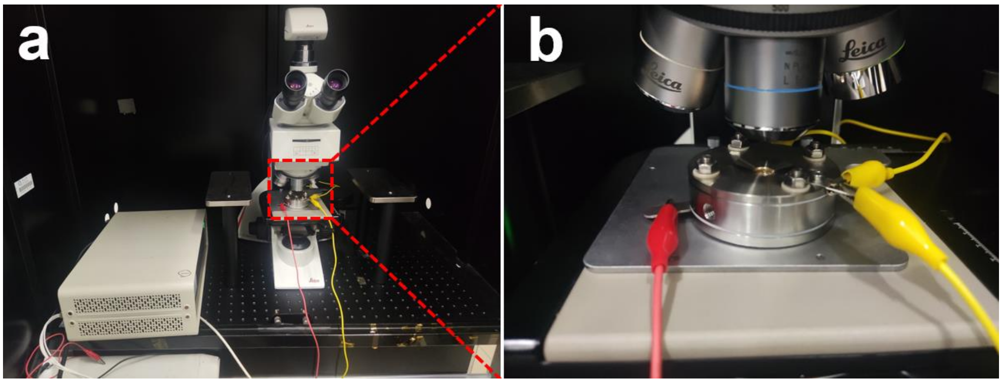

Fig. S2 Photograph of in-situ visualization testing system

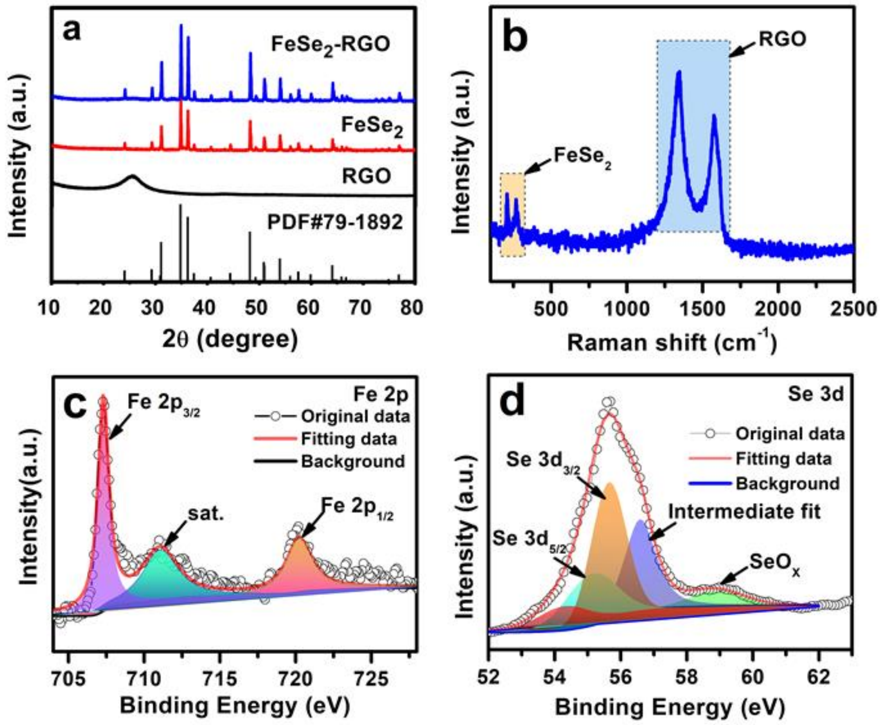

Fig. S3 (**a**) XRD patterns of RGO, $FeSe_2$ and $FeSe_2$@RGO; (**b**) Raman spectrum of $FeSe_2$@RGO; (**c**) Fe 2p and (**d**) Se 3d XPS spectra of $FeSe_2$@RGO

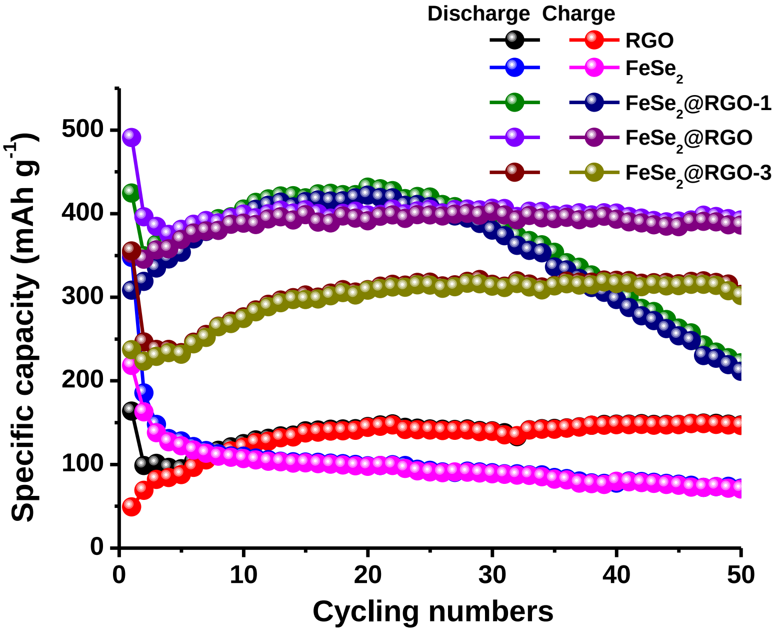

Fig. S4 Cycling performance of RGO, $FeSe_2$, $FeSe_2$@RGO-1, $FeSe_2$@RGO and $FeSe_2$@RGO-3 with DME-based electrolyte at a current density of 100 mA g^{-1}

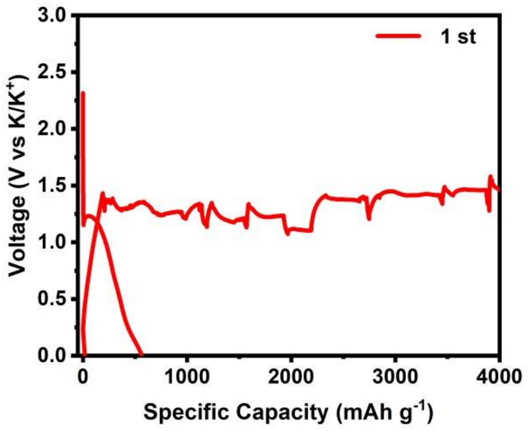

Fig. S5 GCD curves of $FeSe_2$-RGO electrode using DME- 1 based electrolyte

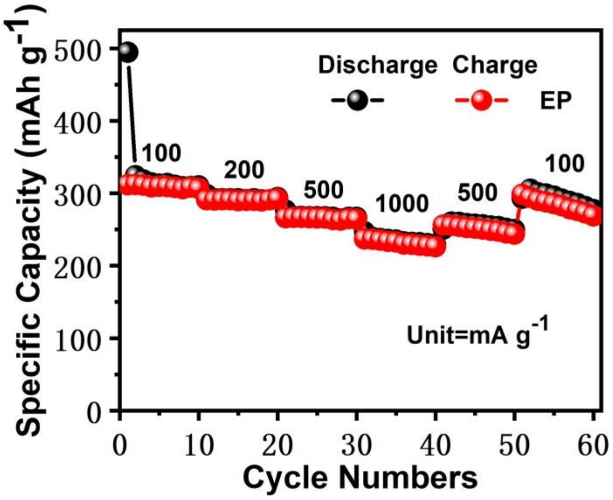

Fig. S6 Rate performance of $FeSe_2$-RGO electrode using EP-based electrolyte

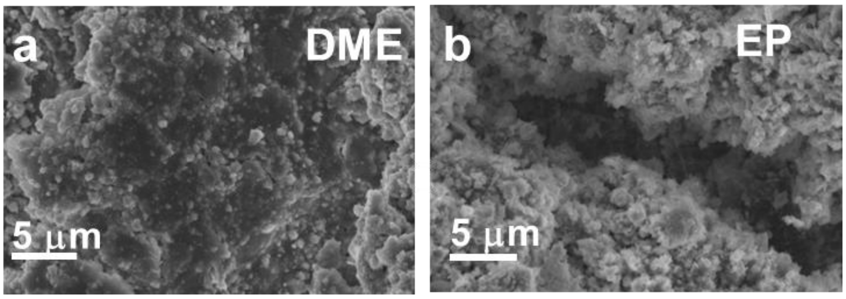

Fig. S7 SEM images of electrode in (**a**) DME-based and (**b**) EP-based electrolytes after cycles

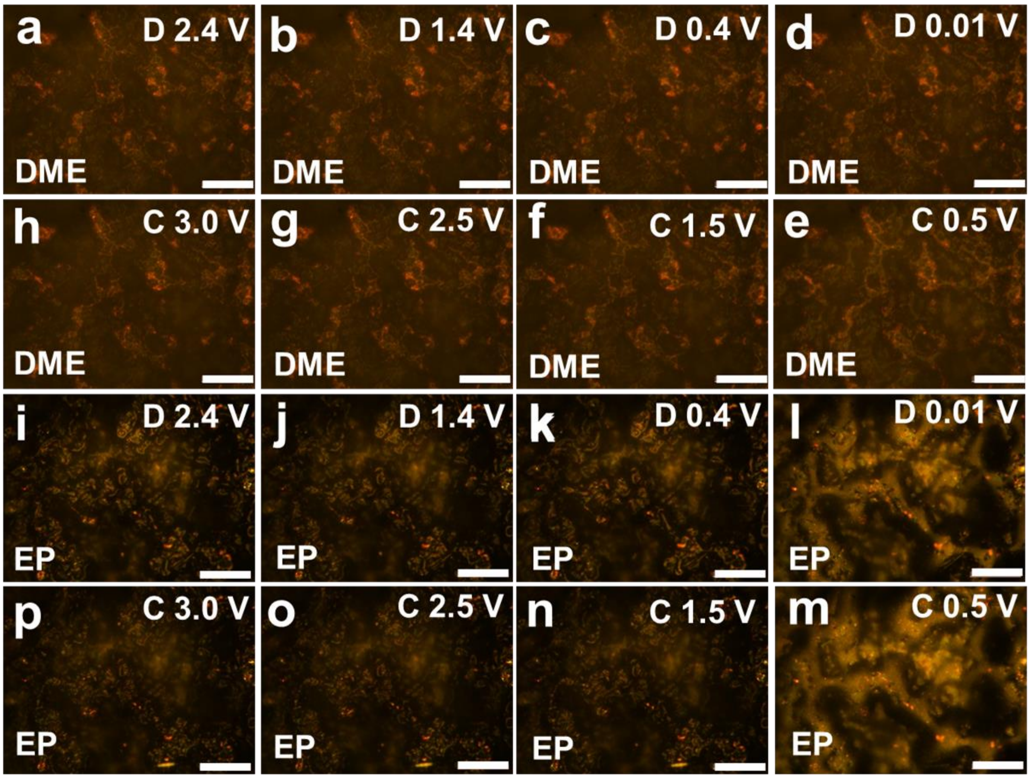

Fig. S8 In situ visualization: (**a-h**) different potassiation-depotassiation states of $FeSe_2$@RGO electrode using DME-based electrolyte in the second cycle; (**i-p**) different potassiation-depotassiation states of $FeSe_2$@RGO electrode using EP-based electrolyte in the second cycle. The scale bar in each image is 50 μm.

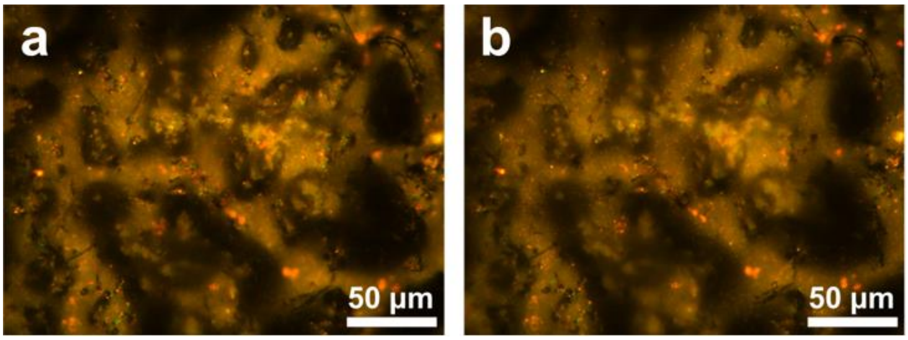

Fig. S9 In situ visualization of $FeSe_2$@RGO electrode using EP-based electrolyte: (**a**) charging to 0.38 V in the first cycle; (**b**) charging to 0.12 V in the second cycle. It is found that huge morphological change in this state, indicating the appearance of inhomogeneous expansion.

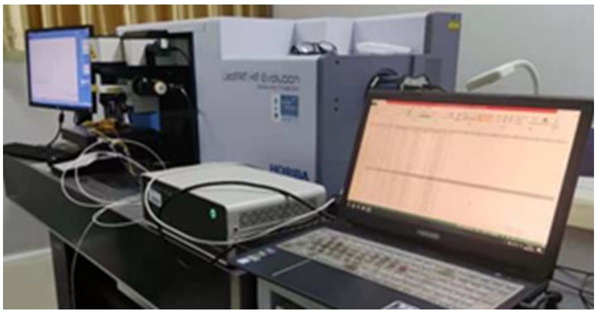

Fig. S10 Photograph of the practical in-situ Raman spectra testing system

Video S1 In-situ visualization of $FeSe_2$@RGO electrode using DME-based electrolyte in initial two cycles.

Video S2 In-situ visualization of $FeSe_2$@RGO electrode using EP-based electrolyte in initial two cycles.
